# Chinese expert consensus on prevention and intervention for elderly with sarcopenia (2023)

**DOI:** 10.1002/agm2.12245

**Published:** 2023-04-26

**Authors:** Hua Cui, Zhaohui Wang, Jianqing Wu, Ying Liu, Jin Zheng, Wenkai Xiao, Ping He, Yun Zhou, Jianye Wang, Pulin Yu, Cuntai Zhang, Jinhui Wu, Geriatrics Branch of the Chinese Medical Association

**Affiliations:** ^1^ Department of Geriatric Cardiology&National Clinic Research Center of Geriatric Diseases Chinese PLA General Hospital Beijing China; ^2^ Department of Geriatrics, Union Hospital, Tongji Medical College Huazhong University of Science and Technology Wuhan China; ^3^ Department of Geriatrics Jiangsu Province Hospital Nanjing China; ^4^ Department of Geriatrics, West China Hospital Sichuan University, China National Clinical Research Center for Geriatric Medicine Chengdu China; ^5^ Beijing Hospital National Center of Gerontology Beijing China; ^6^ Department of Geriatrics, Tongji Hospital, Tongji Medical College Huazhong University of Science and Technology Wuhan China

**Keywords:** elderly, intervention, sarcopenia

## Abstract

Sarcopenia is the age‐related loss of skeletal muscle mass and muscle strength or physical function. It is most common in elderly individuals. Due to its high incidence, insidious onset, and extensive impact on the body, it has a huge impact on the family medical burden and the social public health expenditure in China. The understanding of sarcopenia in China is still lacking, and the recommendations for prevention, control, and intervention are not clear and unified. The purpose of this consensus report is to standardize the prevention, control, and intervention methods for sarcopenia in elderly patients in China; improve the efficacy of intervention; reduce complications during the intervention process; and reduce the risk of falls, fractures, disability, hospitalization, and even death in elderly individuals.

## Graphical abstract

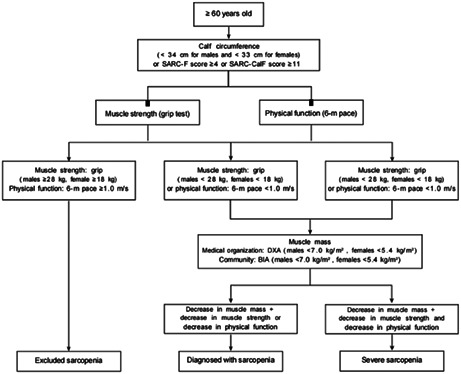

Sarcopenia is the age‐related loss of skeletal muscle mass and muscle strength or physical function. It is most common in elderly individuals. As a common geriatric syndrome, sarcopenia has a huge impact on the family medical burden and the social public health expenditure due to its high incidence, insidious onset, and extensive impact on the body. Research on sarcopenia in China began relatively recently, so the relevant recommendations for prevention, control, and intervention are not clear and unified. To improve its prevention, control, and intervention in the elderly in general hospitals, grassroots medical institutions, nursing homes, and other institutions, this project gathered a number of domestic experts in geriatric‐related disciplines to formulate the present consensus on sarcopenia prevention, control, and intervention in elderly individuals. The writing process of this consensus was as follows: (1) We formed a sarcopenia prevention and intervention expert group, formulated a consensus plan, and drew up a consensus outline. (2) Through an evidence‐based method, we used the Delphi method^1^ for guidance, conducted a literature search and study quality evaluation, synthesized domestic and foreign guideline consensus recommendations, and summarized the published evidence. (3) The project team wrote a consensus based on the evidence and repeatedly organized expert discussions on the content of the consensus. After multiple rounds of feedback and revisions, a consensus was reached. This consensus is suitable for general practitioners in community health service centers, general practitioners in nursing homes, and geriatricians in secondary and tertiary hospitals.

## 1 DEFINITION

While sarcopenia was originally defined solely as a decrease in muscle mass and quantity, research is now placing greater emphasis on the importance of muscle strength, as well as diminished physical function due to the decreased muscle mass. Since 2016, the World Health Organization's International Classification of Diseases (ICD) has officially defined sarcopenia as a disease, code ICD‐10‐CM (M62.84).^2^ A central element of sarcopenia is a decrease in skeletal muscle mass resulting in physical dysfunction. Primary sarcopenia caused by aging and secondary sarcopenia caused by chronic disease or decreased mobility are common in clinical practice. In this consensus, sarcopenia refers to age‐related primary sarcopenia in elderly individuals.

## 2 EPIDEMIOLOGY

The definition, diagnostic criteria, and measurement techniques of sarcopenia have not been uniform, and the backgrounds of the study populations are different, resulting in a large difference in the prevalence of sarcopenia in different studies. The prevalence of sarcopenia in elderly individuals aged 60–70 in the United States is 5% to 13%, and that in individuals aged > 80 years is 11% to 50%.^3^ An epidemiological study in Asian countries using the Asian Working Group for Sarcopenia (AWGS) 2014 criteria showed that the prevalence of sarcopenia was 5.5% to 25.7% and was higher in men than in women (5.1% to 21.0% in men vs. 4.1% to 16.3% in women). In four studies with more than 1000 participants, the prevalence of sarcopenia was 7.3% to 12.0%.^4^ A recent epidemiological survey of sarcopenia in the Chinese population showed that the prevalence of sarcopenia in those aged 60 and above was 5.67% to 23.9%.^5^, ^6^, ^7^, ^8^, ^9^, ^10^, ^11^, ^12^, ^13^, ^14^ There are significant differences in its prevalence in the elderly population by region and sex. It is significantly more common in the eastern region than in the western region^10^, ^11^, ^12^, ^13^, ^14^ and the prevalence rate increases significantly with increasing age.^5^, ^6^, ^7^, ^8^, ^9^ The prevalence rate in the community is lower than that in hospitals and nursing homes,^15^ likewise rural areas over urban areas.^16^

## 3 RISK FACTORS AND PATHOGENESIS

There are many risk factors for sarcopenia. First, it is closely related to aging. The decline in various organ functions and changes in hormone levels in elderly individuals can lead to decreased motor capacity and loss of muscle mass and muscle strength. Second, long‐term bed rest, sedentary behavior, long‐term alcohol drinking and smoking, insufficient dietary energy, protein and vitamins, existing chronic diseases, surgery, malignant tumors, endocrine diseases, multiple‐organ failure, certain drug treatments, and other factors can cause sarcopenia. Among them, primary sarcopenia is only related to age, and secondary sarcopenia is mostly related to exercise, nutrition, and disease.

As research on the etiology of sarcopenia progresses, the following pathogenic hypotheses have become well recognized: α motor neurons and motor units are progressively lost, muscle fibers are denervated, and the surviving motor neurons contact more muscle fibers; skeletal muscle protein synthesis and/or metabolism become unbalanced, mainly due to the abnormal ubiquitin‐proteasome proteolytic system; abnormal mitochondrial function leads to a series of abnormal cell signaling pathways in skeletal muscle cells, resulting in skeletal muscle atrophy and loss, increased apoptosis of skeletal muscle cells, diminished number and function of satellite cells, and increased inflammatory cytokines.^4^, ^17^, ^18^, ^19^

## 4 COMMON ASSESSMENT METHODS

### 4.1 Screening cases

The strength, assistance with walking, rising from a chair, climbing stairs, and falls (SARC‐F) questionnaire or SARC‐F combined with calf circumference (SARC‐CalF) is recommended to use for screening. The recommended calf circumference for sarcopenia screening is <34 cm for men and <33 cm for women^20^; a SARC‐F score ≥4 is considered positive for the screen, and a SARC‐CalF score ≥11 is considered positive for the screen.

### 4.2 Muscle mass assessment

Dual‐energy X‐ray absorptiometry (DXA) is widely used, causes low radiation exposure, can clearly distinguish different tissue components, and can produce reproducible appendicular skeletal muscle mass (ASM) data in a short time. The disadvantage is that DXA devices are not portable and so cannot be widely used in the community, and the measurement results of different DXA devices vary greatly. Muscle mass <7.0 kg/m^2^ in men and <5.4 kg/m^2^ in women by DXA are considered reduced muscle mass.

Bioelectrical impedance analysis (BIA) technology is noninvasive, inexpensive, simple to operate, portable, and rich in functional information. It has often been used in large‐scale population screens in recent years. BIA mainly collects and measures the electrical impedance changes of tissue cells through bioelectric sensors and calculates the individual's fat volume and whole‐body muscle mass, but the accuracy of the results depends heavily on the algorithm the machine uses.^21^ Muscle mass <7.0 kg/m^2^ in males and <5.7 kg/m^2^ in females by BIA is considered decreased muscle mass.

In addition, computed tomography (CT) and magnetic resonance imaging (MRI) are common imaging methods for muscle mass assessment, but their equipment is bulky, immobile, and expensive, and it lacks a measurement threshold for low muscle mass, limiting its practical application for sarcopenia screening.

**Consensus 1**: DXA is the gold standard for measuring muscle mass. BIA is relatively simple and convenient and is more suitable for large‐scale screens and diagnoses in communities and hospitals.

### 4.3 Assessment of muscle strength

There are few accurate and effective methods for measuring muscle strength. It is recommended to use a grip dynamometer to measure grip strength as the first choice for the evaluation and diagnosis of sarcopenia. During the measurement, the left and right hands are measured three times, and the maximum grip strength value is taken. Values of <28 kg for males and <18 kg for females are usually the cutoff values for muscle strength decline. Due to the influence of population and ethnicity, it is suggested that specific cutoff values be determined for each specific population.

When grip strength cannot be measured due to hand trauma, disability, arthritis of the fingers, etc., the five‐time sit‐to‐stand test can be used to record the time from sitting to standing five times as an alternative measure of muscle strength.

**Consensus 2**: Using a grip dynamometer to measure upper‐extremity grip strength is the most commonly used method for assessing muscle strength.

### 4.4 Physical function assessment

The measurement methods of physical function include walking pace, the 6‐min walking test, and the Short Physical Performance Battery (SPPB). The pace test refers to the time required for an individual to walk 4 m or 6 m at a normal pace from the beginning of the movement. This can reflect the individual's physical strength: the faster the speed, the higher the physical fitness level.^22^, ^23^ Since many factors influence the measurement of short‐distance pace in elderly individuals, this consensus recommends using the 6‐meter pace test, with a diagnostic cutoff value of <1.0 m/s.

**Consensus 3**: Use the 6‐m pace test when possible to assess physical function.

## 5 DIAGNOSTIC CRITERIA AND PROCEDURES

**FIGURE 1:**
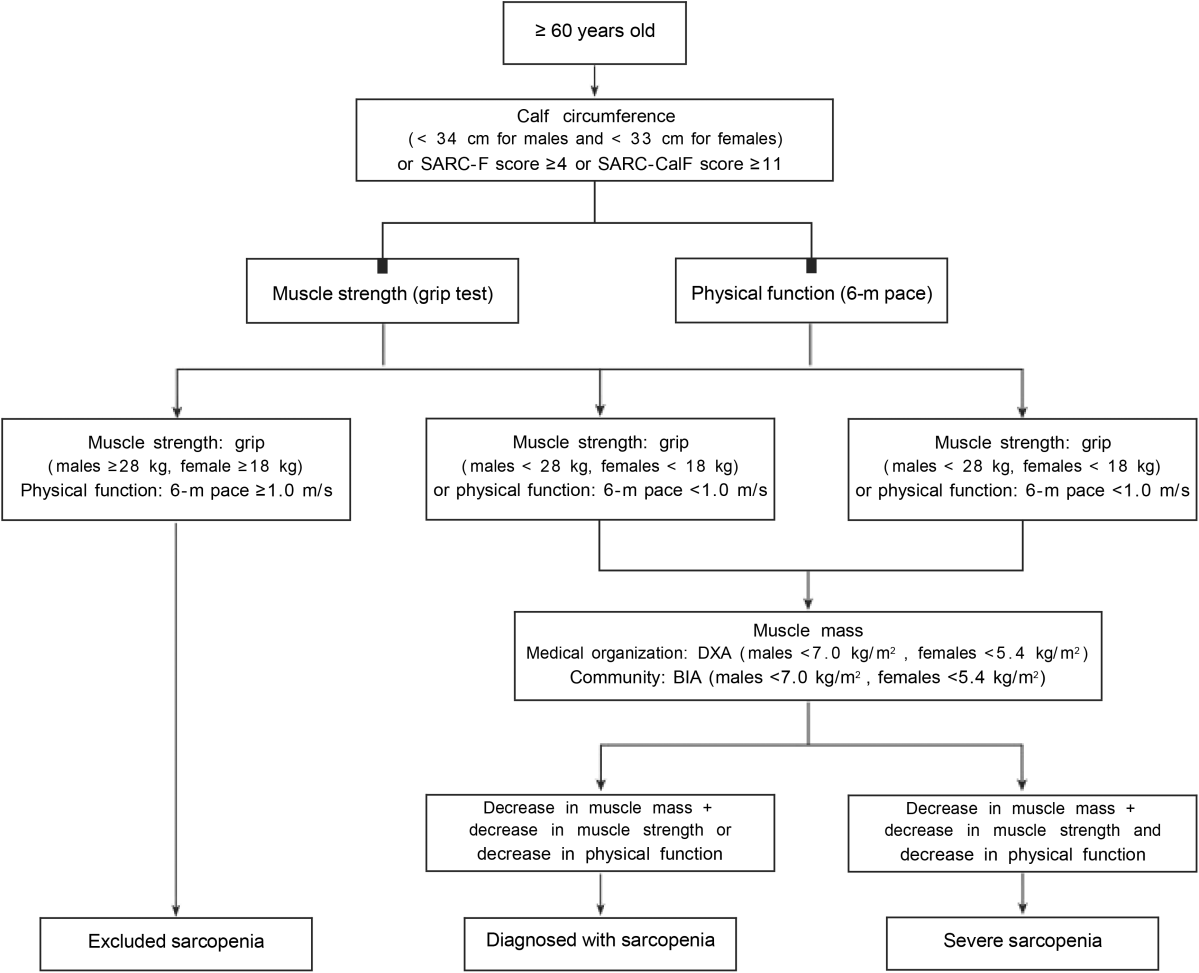
Flow chart of diagnostic criteria for sarcopenia in the elderly. Note: BIA, bioelectrical impedance analysis; DXA, dual‐energy X‐ray absorptiometry; SARC‐CalF, SARC‐F combined with calf circumference; SARC‐F, Strength, assistance with walking, rising from a chair, climbing stairs, and falls questionnaire.

Sarcopenia has only lately garnered much attention in China, and general hospitals have an insufficient understanding of sarcopenia, which has not yet been classified into its own field of study. Sarcopenia is diagnosed when three conditions are met: low muscle mass, muscle strength, and physical function. Low muscle mass is the core element. Considering the difficulty of measuring muscle mass in primary medical institutions, it is recommended to use BIA to measure ASM, and general hospitals can measure it by DXA. Figure 1 lists the detailed diagnostic criteria flowchart.

## 6 INTERVENTION TREATMENT

### 6.1 Exercise intervention

Intervention research on sarcopenia is still in its infancy in China, but it has gradually increased in recent years. Table 1 summarizes 12 randomized controlled studies of exercise intervention for sarcopenia in elderly individuals in China.^24^, ^25^, ^26^, ^27^, ^28^, ^29^, ^30^, ^31^, ^32^, ^33^, ^34^, ^35^ Resistance training–based exercise is generally recommended as the first‐line treatment for sarcopenia. The recommended frequency of the exercise is two to three times/week for 30 min or more, and the training should last at least 12 weeks (two studies lasted 8 weeks).^29^, ^33^ Most domestic studies believe that exercise intervention has a positive effect on improving the appendicular skeletal muscle index (ASMI) of the limbs and can improve the walking speed of elderly patients with sarcopenia. All studies in Table 1 concluded that exercise intervention can improve the muscle strength of elderly patients with sarcopenia, and elderly patients with sarcopenia without obvious exercise contraindications should regularly exercise. The type of exercise intervention recommended is resistance exercise, aerobic exercise, and balance training.^36^, ^37^

**TABLE 1:** Randomized controlled clinical studies of exercise intervention for sarcopenia in elderly individuals in China.

Included literature	Intervention object			Intervention measures			Intervention effect			
	Age (years)	Gender	*n*	Region	Intervention mode	Frequency (times/week)	Time (Week)	Muscle Mass	Muscle strength	Physical function
Liang et al.^1^, ^24^	80–99	Male, female	60	Chengdu	RT, balance	2	12	ASMI*	HS↑	GS↑ SPPB↑
Zhu et al.^25^	≥65	Male, female	113	Hong Kong, China	RT, AT	2	12	ASMI↑	HS↑	GS‐ 5R‐STS**↑**
Wang et al.^26^	60–80	Male, female	60	Shanghai	RT	2–3	12	ASMI‐	HS↑	GS↑ 5R‐STS**↑**
Dong et al.^27^	60–85	Male, female	64	Hunan	RT	3	12	ASMI↑	HS↑	5R‐STS**↑**
Li et al.^28^	≥60	Male, female	241	Beijing Shijiazhuang	RT	3	12	ASMI↑	HS↑	*
Shen et al.^29^	≥65	Male, female	92	Zhejiang	RT, AT, BX, FX	3	8	*	*	*
Wang et al^30^	60–79	Male, female	60	Jiangsu	RT	3 times/week	12	ASMI↑	HS↑	GS↑
Gao^31^	≥ 60	Male, female	60	Hunan	RT, AT	3	24	ASMI*	HS↑	GS↑
Dong^32^	≥60	Male, female	60	Inner Mongolia	RT	3	12	ASMI↑	HS↑	GS↑
Zhang^33^	60–70	Male, female	14	Jilin	RT	3	8	ASMI*	HS↑	GS↑
Wu and Fang^34^	60–95	Male, female	80	Jiangsu	RT	3	48	ASMI↑	HS↑	GS↑
Tang et al.^35^	≥60	Male	200	Sichuan	RT	6	24	ASMI*	HS↑	SPPB↑

*Note*: ↑ means positive effect, − means no significant effect, * means data unknown.

Abbreviations: 5R‐STS, five‐repetition sit‐to‐stand test; ASMI, appendicular skeletal muscle index; AT, aerobic training; BX, balance exercise; FX, flexibility exercise; GS, pace; HS, grip strength; RT, resistance training; SPPB, short physical performance battery.

Sports training venues should be spacious, quiet, ventilated, at a comfortable temperature, well lighted, and equipped with corresponding training equipment, including conventional elastic bands, dumbbells, sandbags, springs, stationary bicycles, and sports bracelets. There should be enough space between all the training equipment to avoid mutual interference. The patient or trainer should bring an intervention record diary, and a designated person should be responsible for recording. There should be relatively sound instruments for measuring vital signs: sphygmomanometer, electrocardiograph, finger oxygen saturation monitor, and material disposal site. There should also be relatively quick access to alcohol, iodophor, gauze, bandage, tape, oxygen inhalation equipment, defibrillator, stretcher, wheelchair, ambulance, etc.

#### 6.1.1 Warm‐up

Three to five minutes of warm‐up exercise should be performed before the main exercise training. Generally, slow walking and joint activities are selected to adjust body function and state, thereby increasing the efficiency of exercise and reducing the likelihood of muscle, ligament, and joint injury during exercise.

#### 6.1.2 Resistance exercise

Resistance exercise is the foundation and core part of exercise intervention. It is characterized by a progressive increase in exercise intensity that enables muscle‐generated forces to move or resist applied resistance.^38^ Resistance training mainly includes the following five aspects: (1) Exercise prescription: resistance training can be divided into three stages: primary, intermediate, and advanced. At the beginning, it is recommended to focus on the familiar resistance training process and precautions stage (1–2 weeks) and gradually progress to the middle and advanced stages. (2) Duration: Each resistance training session is recommended to last 30 to 60 min. There should be at least two to three a week, and the interval between them should be 48 h. (3) Exercise intensity: It is recommended to start with low‐intensity resistance training (40% to 60% of the maximum strength for one repetition (1RM)) in the first 2 weeks. Patients self‐rate their level of exertion during and after exercise. If they reach a score of 12–14 on the Borg Perceived Exertion (RPE) scale, the resistance can be gradually increased by 5%–10% of the 1RM each time. A medium‐ to high‐intensity resistance training program (60% to 80% 1RM) is recommended at the intermediate to advanced level. (4) Number of repetitions and number of sets: It is recommended to repeat each movement in the primary stage 8 to 10 times per set, doing one to two sets with a rest of 1 to 2 min between sets. When it is necessary to increase the intensity of resistance exercise, do more repetitions first, and then increase the training load. (5) Equipment used for exercise: elastic belts, sandbag leggings, dumbbells, etc., can be used to formulate relatively safe weight resistance according to the patient's weight.

#### 6.1.3 Aerobic exercise

Aerobic exercise can improve the cardiopulmonary function and exercise endurance of elderly individuals, improve immunity, enhance the adaptability of the body, and strengthen the adaptation to resistance training, thereby forming a virtuous circle of exercise. Aerobic exercise includes the following: (1) Exercise methods: The most commonly recommended are 6‐min walk, 2‐min high leg lift, and riding a fitness bike. The elderly individuals can also choose traditional Chinese sports and fitness methods, such as fitness dance, *Tai Chi*, *Wu Qin Xi* (five‐animal exercises), and *Baduanjin*. (2) Duration: When done along with resistance training, it is recommended to perform aerobic exercise for 10 to 20 min each time; for aerobic exercise alone, the duration can be extended to 30 to 45 min, at least three times a week. (3) Exercise intensity: The changes in heart rate should be monitored during aerobic exercise, and the heart rate during exercise should be kept at a moderate intensity (50%–80% of the maximum heart rate).^39^ Elderly individuals often have multiple coexisting diseases and take multiple drugs. The results of cardiovascular disease risk assessment, exercise tolerance assessment, and rated perceived exertion scale score should be used as objective reference indices for setting target heart rate.

Another exercise intensity assessment method is to start the patient at an aerobic intensity of two to three metabolic equivalents (METs) and observe their physiological responses, such as heart rate, blood pressure, and fatigue during and after exercise. After adapting to the exercise intensity, under the guidance of the interventionist and according to individual differences, the patient can gradually increase the training intensity of the aerobic exercise.

#### 6.1.4 Balance training

Balance training can help sarcopenia patients maintain body stability in their daily life activities and other activities and reduce the risk of falling. This type of exercise can be divided into the following: (1) Static balance: This refers to the ability to keep the body in a certain posture when the body does not move, such as three‐step balance, standing on one leg, etc. It is recommended that each static movement be held for 10 s at first and gradually increased to 1 to 2 min. (a) The three‐step balance stances are the feet‐together stance, standing with one foot forward and the other foot back much like a walking stance, and standing with the two feet in line underneath the body, one in front of the other, as if on a balance beam. The three postures are performed in sequence. (b) The single‐leg standing training method is to open or close the eyes, put the hands on the hips or the back of a chair, bend one leg, stand on the other foot, and stand while focusing on the soles of the feet. (2) Dynamic balance refers to the ability of the body to stay balanced during exercise, which can be done through sit‐stand‐sit training, walking training, and traditional Chinese fitness methods. (a) Sit‐stand‐sit training: This training helps improve balance while going between sitting and standing in daily life. (b) Walking training: This is conducive to the improvement of pace. It includes straight walking, backwards walking, and sideways walking. (c) Other training: traditional Chinese fitness methods such as fitness dance, *Tai Chi*, five‐animal exercises, *Baduanjin*, etc. During the training process, exercise methods should be adjusted, combined, and exchanged according to the specific situation to avoid psychological and physical fatigue caused by long‐term monotonous exercise training.

#### 6.1.5 Rest and relaxation

After completing a session of resistance training, aerobic training, or balance training, the elderly individuals should walk slowly for 2 min and stretch the major muscle groups and joints to promote blood circulation, which is conducive to the continuous and regular exercise intervention.

#### 6.1.6 Adverse events and treatment

Adverse events during exercise interventions mainly include (1) severe breathing difficulties, profuse sweating, pale complexion, etc.; (2) pain in the precordial area; (3) dizziness or syncope; (4) limb spasm or subjectively severe fatigue and pain; (5) gait imbalance; (6) systolic blood pressure ≥180 mmHg (1 mmHg = 0.133 kPa); (7) a decrease in systolic blood pressure of ≥20 mmHg accompanied by an increased heart rate; (8) blood oxygen saturation (SpO_2_) decreasing and staying lower than 85%; and (9) perceived inability to tolerate the training. The treatment methods include the following: the interventionist asks the patient to stop and rest; monitors vital signs such as blood pressure, heart rate, and SpO_2_; gives emergency treatment on the spot according to the patient's adverse reaction; and sends the patient to hospital for treatment if necessary.

**Consensus 4**: Scientific exercise training for all elderly people aged 60 and above who have been diagnosed with sarcopenia in primary hospitals or general hospitals and have no contraindications to exercise training can effectively improve the relative skeletal muscle index, muscle strength of the limbs, and walking speed.
**Consensus 5**: Aerobic training, resistance training, balance training, and traditional Chinese sports should be combined, and exercise intervention should be combined with nutritional intervention.
**Consensus 6**: Before the first exercise session, a detailed medical history should be taken, any auxiliary examination should be added according to the specific situation, and the patient's condition should be assessed and recorded in the patient's personal file. The benefits, risks, contraindications, and precautions of exercise intervention should be explained to patients and their families before the first exercise, and informed consent should be signed. Vital signs should be measured before each exercise training, and the Borg scale should be used to score the patients' breathing and fatigue before exercise and record them in the exercise training diary.
**Consensus 7**: Blood pressure, heart rate, SpO2, and fatigue during exercise should be monitored and recorded.
**Consensus 8**: After exercise, patients should be asked about their subjective fatigue level; their respiration and fatigue level should be assessed by the Borg scale; their blood pressure, heart rate, and SpO2 should be recorded; and an individualized exercise prescription should be formulated according to each parameter and the presence of adverse reactions.

### 6.2 Exercise intervention programs for special populations

#### 6.2.1 People with cardiopulmonary dysfunction

Before exercising, a professional exercise training doctor should conduct a cardiopulmonary exercise test for patients with cardiopulmonary dysfunction to evaluate the patient's cardiopulmonary function to select appropriate exercise equipment and formulate tolerable exercise intensity, exercise frequency, and duration. Intermittent exercise and progressive training were used, and the patient's vital signs, perceived fatigue level, and dyspnoea scale during and after exercise were recorded to evaluate whether the patient could tolerate the exercise intensity and make corresponding adjustments.

#### 6.2.2 Obese people

It is recommended to increase the frequency, time, and intensity of training and increase the exercise time to 45–60 min/d, 5–7 times/week. The initial resistance training intensity can be appropriately increased by 5% to 10% of the 1RM on the basis of a starting low‐intensity resistance training program (40% to 60% of 1RM) and then gradually increased to high‐intensity resistance training (60% to 80% of 1RM). Training of large muscle groups and total energy consumption should be increased, and training compliance should be improved as much as possible under the premise of ensuring patient training safety.

#### 6.2.3 People with balance disorders

It is recommended to add traditional Chinese sports such as *Tai Chi*, which can effectively improve the balance control ability of the elderly.^40^, ^41^

### 6.3 Nutritional support

Malnutrition is a common geriatric syndrome that can co‐occur with sarcopenia. Malnutrition and the decrease in muscle protein synthesis are important causes and strong predictors of the occurrence and progression of sarcopenia^42^ and important measures for the intervention of sarcopenia.^43^, ^44^ Elderly patients with sarcopenia should use the Mini‐Nutrition Assessment (MNA) to evaluate their nutritional status.^45^ For sarcopenic patients with malnutrition or risk of malnutrition, reasonable nutritional supplements are required while eating freely.

It is recommended that elderly individuals increase their intake of protein containing essential amino acids, such as lean meat and other leucine‐rich foods (such as soybeans, peanuts, etc.).^46^ The daily protein intake of sarcopenia patients should reach 1.2 to 1.5 g/kg/d, of which the proportion of high‐quality protein such as animal protein must reach more than 50%; for sarcopenia patients with severe malnutrition, the daily protein needs to be supplemented to 1.5 g/kg/d or more.^47^, ^48^ The daily protein intake needs to be evenly distributed among the three meals. A balanced distribution can achieve a greater rate of muscle protein synthesis than concentrating it in a single meal.^49^ If additional protein supplementation is needed, it should be done between meals. Whey protein is rich in essential amino acids such as leucine and has a high utilization rate of digestion and absorption. On the basis of daily diet and exercise, supplementing two times a day and ingesting 15 to 20 g of whey protein each time can prevent muscle attenuation in elderly individuals and improve muscle strength.^50^ β‐Hydroxy‐β‐methylbutyric acid (HMB) is a key active metabolite in protein regulation. Resistance training combined with HMB supplementation has preventive and therapeutic effects on sarcopenia in the elderly.^51^ Daily supplementation of 3 g HMB is recommended for elderly patients with sarcopenia, especially if sedentary or bedridden.^52^, ^53^

Low plasma docosahexaenoic acid (DHA) and eicosapentaenoic acid (EPA) concentrations are associated with lower exercise capacity in the elderly.^54^ The use of n‐3 polyunsaturated fatty acids in combination with other nutrients can significantly improve muscle strength and muscle protein synthesis in elderly individuals^55^ and improve exercise capacity.^56^ Daily supplementation with 3000 mg of DHA plus a certain amount of EPA (minimum 800 mg/d) may be needed for these to have a beneficial effect on physical activity in elderly individuals, as doses below this level may not have significant benefits.^57^ Currently, the optimal ratio of DHA to EPA is still unclear.

Vitamin D supplementation can significantly increase muscle strength, especially in patients with serum 25‐hydroxyvitamin D (25(OH)D) concentrations <50 nmol/L or advanced age,^58^, ^59^ while for those without vitamin D deficiency, there is no clear improvement in mobility with additional supplementation. Therefore, routine vitamin D supplementation is not recommended in elderly patients with sarcopenia. It is more meaningful to guide vitamin D supplementation in combination with the serum 25(OH)D concentration of patients. When serum 25(OH)D < 50 nmol/L, supplementation can be given. The usual dose of vitamin D supplementation is 15 μg/d (600 U/d) for people under the age of 70 and 20 μg/d (800 U/d) for those 70 years and older.^60^ D2 and D3 have the same effect at maintaining 25(OH)D levels.^61^

Dietary intervention alone usually cannot provide enough nutrients for elderly patients with sarcopenia. On the basis of a recommended dietary intervention, when sarcopenia patients eat less than 80% of the recommended target amount (20–30 kcal/kg/d), oral nutritional supplementation (ONS) is recommended.^62^ ONS can effectively prevent sarcopenia in frail elderly individuals and improve their muscle mass, strength, and exercise capacity.^63^ The intake of ONS preparations is 400 to 600 kcal/d, which should be taken between meals or after exercise or sipped at 50 to 100 mL/h, and ONS preparations with different nutritional formulas should be selected according to the patient's comorbidities.

Nutritional supplementation combined with exercise intervention is a powerful measure to maintain muscle function.^64^ Studies on sarcopenia in elderly individuals in China have confirmed that after 12 weeks of intensive lifestyle intervention (including nutritional supplementation + resistance training), the muscle mass of the patients was significantly improved, and the inflammatory indices were reduced.^65^ Therefore, the combination of nutritional intervention and exercise in elderly patients with sarcopenia is advocated.

**Consensus 9**: It is recommended that elderly patients with sarcopenia be screened for nutritional risk and given active nutritional supplementation, especially adequate protein/essential amino acid supplementation. Oral nutritional supplementation in sarcopenic patients with malnutrition can help improve muscle mass and muscle strength in sarcopenic patients.
**Consensus 10**: The recommended intake of protein for elderly patients with sarcopenia is 1.2 to 1.5 g/kg/d, and the proportion of high‐quality protein should preferably reach 50%, which should be evenly distributed into three meals a day.
**Consensus 11**: Routine supplementation with vitamin D is not recommended in elderly patients with sarcopenia. It is more meaningful to guide vitamin D supplementation based on the serum 25(OH)D concentration. When serum 25(OH)D < 50 nmol/L, supplements can be given.
**Consensus 12**: A comprehensive preventive measure combining nutritional supplementation and exercise intervention is recommended.

### 6.4 Drug treatment

To date, there is insufficient evidence for drug treatment of sarcopenia, and there is no recommended first‐line drug for sarcopenia in clinical practice. There has been much research on drugs for sarcopenia in the past, but most drugs only improve skeletal muscle mass and have no effect on physical functions such as muscle strength and pace. Current drugs for the treatment of sarcopenia mainly include selective androgen receptor modulators, myostatin, and an antagonist for the activin type II receptor pathway. A review of pharmacotherapy for sarcopenia included clinical trials of 10 drugs, of which only vitamin D (especially in older women) and testosterone (in older men with clinical muscle weakness and low serum testosterone) improves muscle mass, muscle strength, and/or physical performance, and there is no evidence that other pharmacological interventions are effective.^66^

### 6.5 Traditional Chinese medicine

#### 6.5.1 Chinese traditional sports

(1) *Tai Chi*: *Tai Chi* is slow and rhythmic comprehensive bodily movements that include postural adjustment, weight transfer, and coordination with breathing. *Tai Chi* is recommended as the preferred balance training method by American and British geriatric societies.^67^, ^68^ Simplified 24‐form *Tai Chi* is recommended for elderly individuals with sarcopenia. The simplified 24‐form *Tai Chi* is simple and easy to learn and remember, with gentle movements that are highly safe. Long‐term adherence can improve the muscle strength for flexion and extension of the knee and ankle joints of elderly individuals, as well as lower‐extremity proprioception and sensitivity. It can also increase postural control, cardiorespiratory fitness, and lower extremity muscular endurance, thereby reducing the risk of falls in older adults.^69^, ^70^, ^71^, ^72^, ^73^ It is recommended as a balance training program for elderly individuals and a routine rehabilitation therapy and regular physical exercise program for the elderly in the community. There are a total of 24 movements in the simplified 24‐form Tai Chi, and it takes 5 to 8 min to complete one movement after becoming proficient. It is recommended to do each exercise two to three times, with 3 to 5 min of rest between each time, and to do this 3 to 5 days a week for more than 12 weeks. (2) *Wu Qin Xi*: The health *qigong* known as *Wu Qin Xi* is relatively simple and easy to learn. It does not have high requirements for the venue, equipment, or intervention instructor. It can effectively improve the balance ability, lower limb muscle strength, gait, cardiopulmonary function, and quality of life of elderly patients with sarcopenia.^74^, ^75^ It is recommended to start with a 1‐week study period. After mastering it, train three to five times a week, each lasting 30 to 60 min. The complete movement can be done two to three times, with a rest of 3 to 5 min between sets. It is recommended to stick to 12 weeks at first. (3) *Baduanjin*: The new health *qigong* named *Baduanjin* can improve the balance of the elderly and reduce the proportion of body fat and blood lipid levels. It plays a systematic role in exercising the bones, ligaments, spine, joints, and cardiopulmonary functions of elderly individuals.^76^, ^77^ The steps are simple and easy to operate, the overall action rhythm is soothing, the exercise intensity is controllable, and the requirements for venues, equipment, and intervention instructors are not high, which meets the needs of traditional fitness training for elderly individuals in China. Therefore, *Baduanjin* can be used as an intervention program for people with sarcopenia, a rehabilitation program for people with cardiopulmonary dysfunction, and a form of daily exercise for the healthy elderly population in China. *Baduanjin* has eight movements, and they require a 1‐week study period. It is recommended to train three to five times a week for 30 to 60 min each time. The complete movements can be practiced two to three times each day.

#### 6.5.2 Traditional Chinese medicine

Sarcopenia belongs to the category of “atrophic disease” in traditional Chinese medicine, and the disease is located in the tendon, vessel, and muscles. According to traditional Chinese medicine theory, the spleen controls the muscles, and it is believed that the etiology of sarcopenia is mostly indigestion caused by spleen deficiency and lack of muscle movement caused by insufficient nutrient intake.^78^ The clinical treatment of this disease in modern Chinese medicine mainly focuses on nourishing the spleen and stomach. Drugs with the function of nourishing the spleen and *qi* can improve the antioxidant capacity of mitochondria, reduce the damage to skeletal muscle, and delay the occurrence and progression of sarcopenia.^79^ Several prescriptions that mainly regulate the spleen and stomach (Bazhen decoction, Buzhong Yiqi decoction, Sijunzi decoction, etc.) combined with nutritional support and exercise have significantly improved sarcopenia patients' muscle mass, strength, function, and activities of daily living.^80^, ^81^, ^82^ However, research on traditional Chinese medicine treatment of sarcopenia is still in its infancy, and it is worthy of further attempts and discussion.^83^

Expert group (in alphabetical order of surname): Jiumei Cao (Department of Geriatrics, Ruijin Hospital Affiliated to Shanghai Jiaotong University School of Medicine); Bo Chen (Department of Geriatrics, Jiangsu Province Hospital); Zheng Chen (Department of Geriatrics, Beijing Geriatric Hospital); Hua Cui (Department of Cardiology, Second Medical Center of the Chinese People's Liberation Army General Hospital); Shuiping Dai (Geriatrics Center, West China Hospital, Sichuan University); Linzi Deng (National Center of Gerontology, Beijing Hospital); Huan Feng (Department of General Medicine, Third Hospital of Mianyang); Jinglong Gao (Geriatrics Hospital of Shaanxi Provincial People's Hospital); Xuewen Gao (Geriatrics Institute of Inner Mongolia Autonomous Region People's Hospital); Wen Guo (Geriatrics Center, West China Hospital, Sichuan University); Ping He (Department of Geriatrics, Union Hospital, Tongji Medical College, Huazhong University of Science and Technology); Song Hu (Department of Geriatrics, The Affiliated Hospital of Qingdao University); Lin Kang (Department of Geriatrics, Peking Union Medical College Hospital); Feika Li (Department of Geriatrics, Ruijin Hospital Affiliated to Shanghai Jiaotong University School of Medicine); Rui Li (Geriatric Neurology, Shaanxi Provincial People's Hospital); Siyuan Li (Geriatrics Center, West China Hospital, Sichuan University); Yan Li (Geriatric Medicine Department, Yunnan Provincial First People's Hospital); Gongxiang Liu (Geriatrics Center, West China Hospital, Sichuan University); Lina Ma (Department of Geriatric Diseases, Xuanwu Hospital, Capital Medical University); Xunlong Ma (Department of General Medicine, Third Hospital of Mianyang); Yongjun Mao (Department of Geriatrics, The Affiliated Hospital of Qingdao University); Li Mo (Geriatrics Center, West China Hospital, Sichuan University); Xiushi Ni (Department of Geriatrics, Shanghai General Hospital, Shanghai Jiaotong University); Huiyun Pan (Geriatrics Center, the First Affiliated Hospital, Zhejiang University School of Medicine); Mingzhao Qin (Cadre Medical Department, Beijing Tongren Hospital, Capital Medical University); Juan Song (Geriatrics Center, West China Hospital, Sichuan University); Yuetao Song (Beijing Geriatrics Hospital Geriatric Health and Integrated Medical Care Research Office); Xiaohong Sun (Department of Geriatrics, Peking Union Medical College Hospital); Zhe Tang (Xuanwu Hospital, Capital Medical University); Fangyuan Tian (Department of Clinical Pharmacy, West China Hospital, Sichuan University); Yingxuan Tian (Department of Respiratory, Shanxi Provincial People's Hospital); Zhaohui Wang (Department of Geriatrics, Union Hospital, Tongji Medical College, Huazhong University of Science and Technology); Jiahe Wang (Department of General Medicine, Shengjing Hospital of China Medical University); Jianye Wang (National Center of Gerontology, Beijing Hospital); Qing Wang (Department of Geriatrics, Fuxing Hospital of Capital Medical University); Fang Wu (Department of Geriatrics, Ruijin Hospital Affiliated to Shanghai Jiaotong University School of Medicine); Jianqing Wu (Department of Geriatrics, Jiangsu Province Hospital); Jinhui Wu (Geriatrics Center, West China Hospital, Sichuan University); Huan Xi (National Geriatrics Center of Beijing Hospital); Wenkai Xiao (Department of Cardiology, Second Medical Center of Chinese People's Liberation Army General Hospital); Ming Yang (Geriatrics Center, West China Hospital, Sichuan University); Pulin Yu (National Center of Gerontology, Beijing Hospital); Cuntai Zhang (Department of Geriatrics, Tongji Hospital, Tongji Medical College, Huazhong University of Science and Technology); Shaomin Zhang (Geriatrics Center, West China Hospital, Sichuan University); Jin Zheng (Department of Cardiology, Second Medical Center of Chinese People's Liberation Army General Hospital); Baiyu Zhou (National Center of Gerontology, Beijing Hospital); Yun Zhou (Department of Geriatrics, Union Hospital, Tongji Medical College, Huazhong University of Science and Technology).

## AUTHOR CONTRIBUTIONS

Initiate and organization of this consensus: Jinhui Wu. Writing the initial draft (including substantive translation): Hua Cui, Zhaohui Wang, Jin Zheng, Wenkai Xiao, Ping He, Yun Zhou. Preparation and presentation of the published work: Jianqing Wu, Ying Liu. Critical review and revision: Jianye Wang, Pulin Yu, Cuntai Zhang, Geriatrics Branch of the Chinese Medical Association, and the Expert Group of the Chinese Expert Consensus on Prevention and Intervention for Elderly with Sarcopenia.

## FUNDING INFORMATION

National Key R&D Program of China (2018YFC2002100, 2018YFC2002102, 2018YFC2002103)

## CONFLICT OF INTEREST STATEMENT

Jianye Wang, Pulin Yu, Cuntai Zhang are the Editorial Board members of Aging Medicine and co‐authors of this paper. To minimize bias, they were excluded from all editorial decision making related to the acceptance of this paper for publication. Other authors have nothing to disclose.

## ETHICS STATEMENT

Not applicable.
